# Insect Odorant Response Sensitivity Is Tuned by Metabotropically Autoregulated Olfactory Receptors

**DOI:** 10.1371/journal.pone.0058889

**Published:** 2013-03-12

**Authors:** Merid N. Getahun, Shannon B. Olsson, Sofia Lavista-Llanos, Bill S. Hansson, Dieter Wicher

**Affiliations:** Department Evolutionary Neuroethology, Max Planck Institute for Chemical Ecology, Jena, Germany; Plant and Food Research, New Zealand

## Abstract

Insects possess one of the most exquisitely sensitive olfactory systems in the animal kingdom, consisting of three different types of chemosensory receptors: ionotropic glutamate-like receptors (IRs), gustatory receptors (GRs) and odorant receptors (ORs). Both insect ORs and IRs are ligand-gated ion channels, but ORs possess a unique configuration composed of an odorant-specific protein OrX and a ubiquitous coreceptor (Orco). In addition, these two ionotropic receptors confer different tuning properties for the neurons in which they are expressed. Unlike IRs, neurons expressing ORs are more sensitive and can also be sensitized by sub-threshold concentrations of stimuli. What is the mechanistic basis for these differences in tuning? We show that intrinsic regulation of Orco enhances neuronal response to odorants and sensitizes the ORs. We also demonstrate that inhibition of metabotropic regulation prevents receptor sensitization. Our results indicate that Orco-mediated regulation of OR sensitivity provides tunable ionotropic receptors capable of detecting odors over a wider range of concentrations, providing broadened sensitivity over IRs themselves.

## Introduction

Insects, for which olfaction is of primary importance for survival [1], [2] possess remarkable chemosensory capabilities. Male silkworm moths, for example, are able to respond behaviourally to 3000 molecules/ml air [3]. Nevertheless, the cellular and molecular mechanisms underlying the outstanding sensitivity of the insect olfactory system are not well understood.

Insects are known to possess three different types of chemosensory receptors: odorant receptors (ORs), ionotropic glutamate-like receptors (IRs), and gustatory receptors (GRs) [4]–[6]. IRs are three-transmembrane proteins, whereas GRs and ORs are seven-transmembrane proteins [5]–[7]. Insect odorant receptors (ORs) also exhibit a unique configuration of heterodimers composed of an odorant-specific olfactory receptor protein (OrX) and a ubiquitous coreceptor (Orco) [7] which operate as ligand-gated ion channels [8], [9].

The independent evolution [10], [11] of these two different ionotropic receptor families (ORs/GRs and IRs) has become a great topic of speculation for the field (e.g. [2], [12]). Why do these multiple families persist among all higher insect orders? And why do they possess such radically different molecular conformations? Initially, it was suggested that these multiple families expand the affinity of the olfactory palette to different chemical classes [6], [13]–[15]. However, a recent study also revealed that olfactory sensory neurons (OSNs) expressing ORs, GRs, or IRs exhibit intrinsic differences in temporal kinetics to brief or intermittent stimuli [16]. Specifically, OR-expressing neurons respond faster and with higher sensitivity to brief stimulation, while IR-expressing neurons do not adapt to long stimulations. This implies that OR-expressing neurons are more accurate at detecting the low-concentration, punctate plume packets received at long distances from the odor source [17], while IR-expressing neurons can better track the high-concentration, long lasting stimulation received when on or near the source [16]. This diversity offers both broader ligand specificity and expanded spatiotemporal dynamics with which to parse the odor world, and is particularly important for insects challenged by the high-speed performance of flight [16]. Interestingly, the purported evolution of ORs [11], [18] corresponds well to the evolution of flight during the Carboniferous Era (see [19]).

Given that ORs appear to offer mechanistic differences to IRs (c.f. [12], [20]), what aspects of the OR molecular structure and/or function generate these advantages? Indeed ORs are ionotropic receptors, although their inverted 7-transmembrane topology is considerably different in structure to the 3-transmembrane IRs. In addition, the involvement of G proteins in the olfactory signal transduction of insect ORs remains controversial [21]–[23]. In heterologously expressed insect ORs, ligand application elicited a fast ionotropic current [8], [9] that was accompanied by a slow, metabotropic current. Ligand binding to OrX led to enhanced cAMP production and activated an ion channel formed by the Orco protein [9]. We previously demonstrated that activators of phospholipase C (PLC) or protein kinase C (PKC) can stimulate Orco channel activity, while inhibition of PLC or PKC abolishes Orco sensitivity to cAMP [24].

Given the relatively low sensitivity exhibited by ionotropic receptors alone [16], might this suggested metabotropic activity contribute to the high olfactory sensitivity of insect ORs? To address this question, we combined extracellular recording of OSN activity upon odor stimulation with simultaneous microinjection of compounds affecting metabotropic signalling [25]. This technique has been shown to mimic results obtained with in vitro manipulation of second messenger pathways [24], [25]. We also address whether manipulation of the metabotropic pathway affects OSN sensitivity, response range, or sub-threshold sensitization of the neuron to repeated odorant stimulation. Finally, using a genetically manipulated fly with impaired Orco function we independently demonstrate the intrinsic nature of intracellular signaling for sensitizing ORs.

## Materials and Methods

### Extracellular Single Sensillum Recording and Microinjection

Recording and injection protocols performed on *Drosophila melanogaster* flies were as described [25]. 2–5 day old adults were fixed dorsally to a microscope slide [26], [27]. For odor stimulation 10 µl of appropriate concentration was pipetted onto approximately 1 cm filter paper in disposable Pasteur pipettes. Charcoal-filtered and humidified air (approximately 1 l/min) passed over the antenna from a stimulus air controller (Syntech, CS-5, Hilversum, NL) through an aluminium tube approximately 10 mm from the antenna. During stimulation, airflow bypassed a complementary air stream (0.5 l/min during 0.5 s) through the stimulus pipette placed roughly 3 cm from the preparation. Compounds and concentrations for injection were diluted in saline [28] as follows: 8-br-cAMP (1 mM), U73122 (0.5 mM), Gö6976 (0.5 mM), SQ22536 (20 mM), OAG (0.1 mM), PMA (0.1 mM). Note that due to a dilution effect, concentrations of injected agents were 100x the concentration used in isolated cell preparations [25]. To check whether the injected compounds reach the outer OSNs dendrites where the ligand-receptor interaction occurs, we injected the Or22a agonist ethyl butyrate (Etb) at threshold concentration (−9 v/v) into the base of ab3 sensilla. During the 200 s injection period, Etb enhanced the spontaneous activity of the ab3A neuron expressing Or22a, but there was no change in activity for the ab3B neuron (Fig. S1A). To exclude mechanical artifacts that may affect OSNs during long lasting injection, we also tested the effect of saline and 8-br-cAMP microinjection which did not change OSN spontaneous activity over the 300 s recording period (Fig. S1B).

Recordings were performed in Or22a-GAL4; UAS-CD8-GFP flies expressing membrane tagged GFP in 22a-OSNs, and in flies whose endogenous Orco was replaced either with Orco or Orco mut in all Ors expressing OSNs.

Responses were analyzed between 500 and 1350 ms after stimulus onset, accounting for mechanical stimulus delay (150 ms). For response kinetics, spike frequency ratios were analyzed as peri-stimulus time histograms (PSTHs) in 25 ms bins by dividing each 25 ms frequency by the average pre-stimulus frequency over 2 s to give a normalized ratio for each time point. The PSTHs presented in the figures show the normalized means ± standard error of mean (s.e.m.) for *n* cells. Areas under the PSTH curve were measured for each response profile using the trapezoid rule and divided by the time to establish a normalized frequency average for each response.

### Orco Mut and Transgenic Flies

#### Molecular biology and fly genetics

The Orco phosphorylation mutant “Orco mut” was generated as described for “Orco PKC” in [24]. Full-length Orco PKC (now named Orco mut) was digested from Orco PKC-pcDNA3.1(+) and subcloned into pUAST [29] using matching restriction sites. *Drosophila melanogaster* UAS-Orco mut transformants were generated at Aktogen Ltd (University of Cambridge, UK). Two independent lines were used in our experiments (UAS-Orco mut(1) and UAS-Orco mut(2)) with identical results. We generated Orco homozygote null mutant flies (Orco1) expressing either Orco mut (UAS-Orco mut(1) or UAS-Orco mut(2)) or Orco wild-type (UAS-Orco) in Orco22a OSNs (Or22a-Gal4). Control flies were Orco1 homozygote null mutant carrying UAS-Orco mut or UAS-Orco wild type insertions, but no Or22a-Gal4 driver. Antennae mRNA expression was confirmed by RT-PCR and *in situ* hybridization with specific primers and antisense digoxigenin-labeled RNA probe corresponding to Orco cDNA, respectively (not shown). Specific genotypes of flies used in this study were “no Orco”: w/w; +/UAS-Orco mut; Orco1/Orco1; “Orco”: w/w; UAS-Orco/UAS-Orco; Orco1-Or22a-GAL4/Orco1-Or22a-GAL4; “Orco mut”: w/w; UAS-Orco mut/UAS-Orco mut; Orco1-Or22a-GAL4/Orco1-Or22a-GAL4.

#### Insect strains

*Drosophila* stocks were maintained on conventional cornmeal-agar-molasses medium under a 12 h light: 12 h dark cycle at 18°C or 25°C. Mutant alleles and transgenic lines used were: Or22aGAL4; UAS-CD8mGFP (Silke Sachse), Orco1, Orco2 (Bloomington Stock center, [30]), Orco-GAL4 (Bloomington Stock center, [30]), UAS-OrcoPKC(1), UAS-OrcoPKC(2) (this reference).

#### Immunofluorescences

Antennae sections were immunolabeled with primary antibodies against *Drosophila* Orco (1∶1000) and Or22a (1∶100) ([31]; kindly provided by Leslie Vosshall), and secondary anti-antibody conjugated to Alexa Fluor 568 (1∶200, Invitrogen). Confocal images were obtained at 1-µm intervals over 20 µm Z-stack using a LSM510 Meta confocal microscope (Zeiss, Jena, Germany).

### Data Presentation and Statistics

Results were given as means ± standard error of mean (s.e.m.), *n* = number of cells. The evaluation of statistical significance of differences was performed with two-way ANOVA for testing two variables. Mann-Whitney U tests (between treatments) and paired Wilkoxon Signed Ranks tests (within-treatment) compared responses using summary statistics calculated from areas under the peri-stimulus time histogram curve [26] using PASW (SPSS) v. 18 software.

### Chemicals

All odors were purchased from Sigma (Taufkirchen, Germany). Ethyl acetate (Eta, >99%), ethyl butyrate (Etb, 99%), and methyl acetate (Mea, >98%) were dissolved in hexane (99%, Fluka Analytical, Buchs, Switzerland). Phenyl acetaldehyde (PAA >90%) and 1-hexanol (>99%) were diluted in mineral oil (BioChemika Ultra, Fluka); butyric acid (Ba, >99%) and 1,4-diaminobutane (Dab, >98%) were dissolved in water.

8-bromo-cAMP, forskolin, phorbol 12-myristate 13-acetate (PMA), and 9-(tetrahydro-2-furanyl)-9H-purin-6-amine (SQ22536) were obtained from Sigma; U73122, and Gö6976 from Calbiochem (Darmstadt, Germany); 1-oleoyl-2-acetyl-sn-glycerol (OAG) from Alexis (Lörrach, Germany).

## Results

### Repetitive Subthreshold Odor Stimulation Sensitizes ORs but not IRs

**Figure 1:**
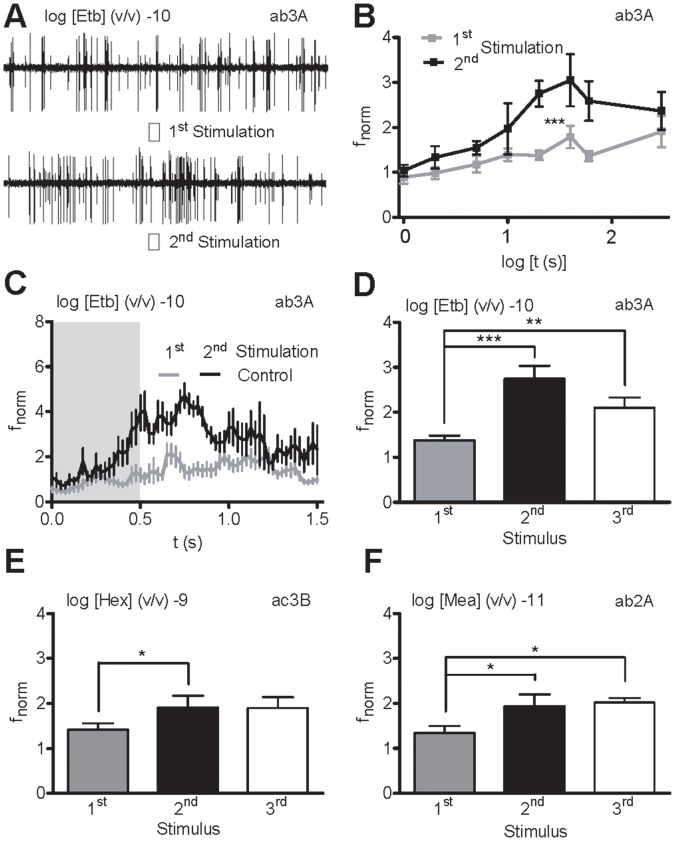
Repeated subthreshold stimulation sensitizes odorant receptors. **A**, Recordings of neuronal activity from ab3 sensilla (large action potentials, ab3A neuron expressing Or22a; small action potentials, ab3B neuron expressing Or85b) upon before and after 20 s repeated ethyl butyrate (Etb) stimulation (−10 v/v; 0.5 s, shaded area). The first stimulation fails to elicit a response while the second does so. **B**, Dependence of normalized ab3A neuron spike frequency (f_norm_) upon 1^st^ and 2^nd^ subthreshold Etb stimulation (−10 v/v; 0.5 s) on the interval between stimulations (*n* = 12). **C**, Time course of f_norm_ for 1^st^ and 2^nd^ stimulation (interval 20 s, *n* = 12). **D–F**, Mean f_norm_ for ab3A (**D**), ac3B (**E**) and ab2A (**F**) neuron to repetitive subthreshold Etb (**D**), ethyl acetate (Eta, **E**) and methyl acetate (Mea, **F**) stimulations (interval 20 s, *n* = 12). *P<0.05, **P<0.01, ***P<0.001; Paired Wilcoxon Signed Ranks test.

We inserted a glass pipette microelectrode into the base of large basiconic ab3 sensilla housing OSNs ab3A expressing the receptor protein Or22a, previously characterized in cultured cells [9] and stimulated the animal with the Or22a ligand [32], ethyl butyrate (Etb). While an initial application of Etb at subthreshold concentration (log −10 dilution) failed to increase OSN activity (Fig. 1A, B), a second or third stimulation presented after at least 10 seconds produced significant odorant responses (Fig. 1A–C). With a 3 min interstimulus period, this sensitization was absent (Fig. 1B). Sensitization by repeated subthreshold odor stimuli were also observed in OSNs ac3B and ab2A expressing Or35b and Or59b, respectively (Fig. 1E, F), as well as in ab1A expressing Or42b (not shown).

**Figure 2:**
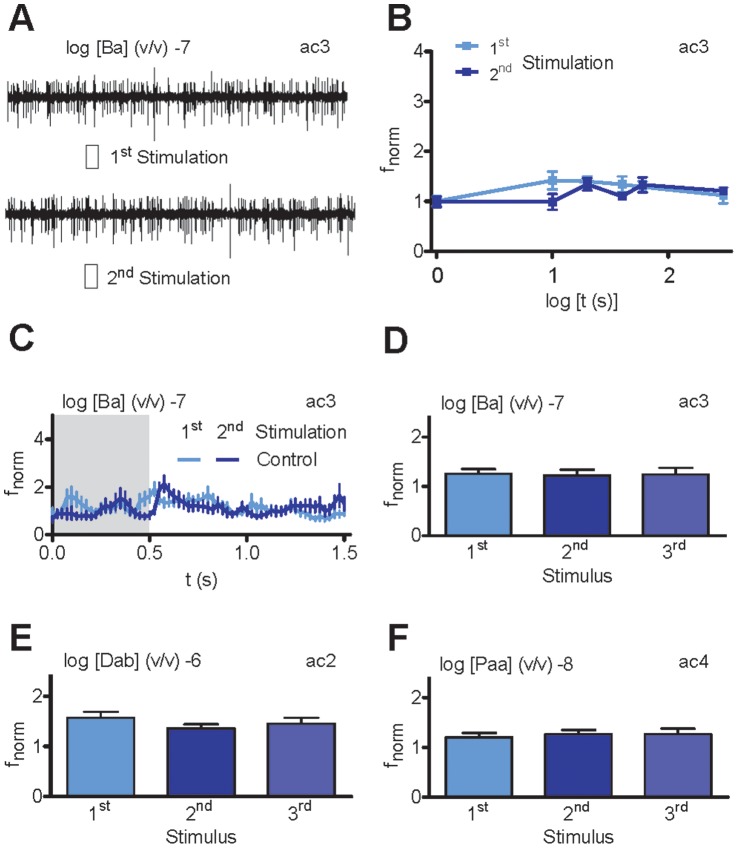
Repeated subthreshold stimulation does not sensitize ionotropic receptors (IRs). **A**, Recordings of neuronal activity from ac3 sensilla (large action potentials, Ir75abc neuron; small action potentials, Or35a neuron) upon before and after 20 s repeated butyric acid (Ba) stimulation (−7 v/v; 0.5 s, shaded area). Both stimulations fail to elicit a response. **B**, Dependence of normalized Ir75abc neuron spike frequency (f_norm_) during 1^st^ and 2^nd^ subthreshold Etb stimulation (−7 v/v; 0.5 s) on the interval between stimulations (*n* = 12). **C**, Time course of f_norm_ for 1^st^ and 2^nd^ stimulation (interval 20 s, *n* = 12). **D–F**, Mean f_norm_ for Ir75abc (**D**), Ir41a (**E**) and Ir84a (**F**) neuron to repetitive subthreshold Ba (**D**), Dab (**E**) and Paa (**F**) stimulations (interval 20 s, *n* = 12). N.s.; Paired Wilcoxon Signed Ranks test.

However, repetitive subthreshold stimulation of ac3 OSNs expressing Ir75abc did not lead to an increased response after a second or third stimulation for interstimulus intervals ranging from 10 s to 3 min (Fig. 2A–D). In addition, ac2 and ac4 OSNs expressing Ir41a and Ir84a, respectively, could not be sensitized by repeated stimulation (Fig. 2E, F).

### Metabotropic Signalling Shapes the Odorant Response of OSNs

**Figure 3:**
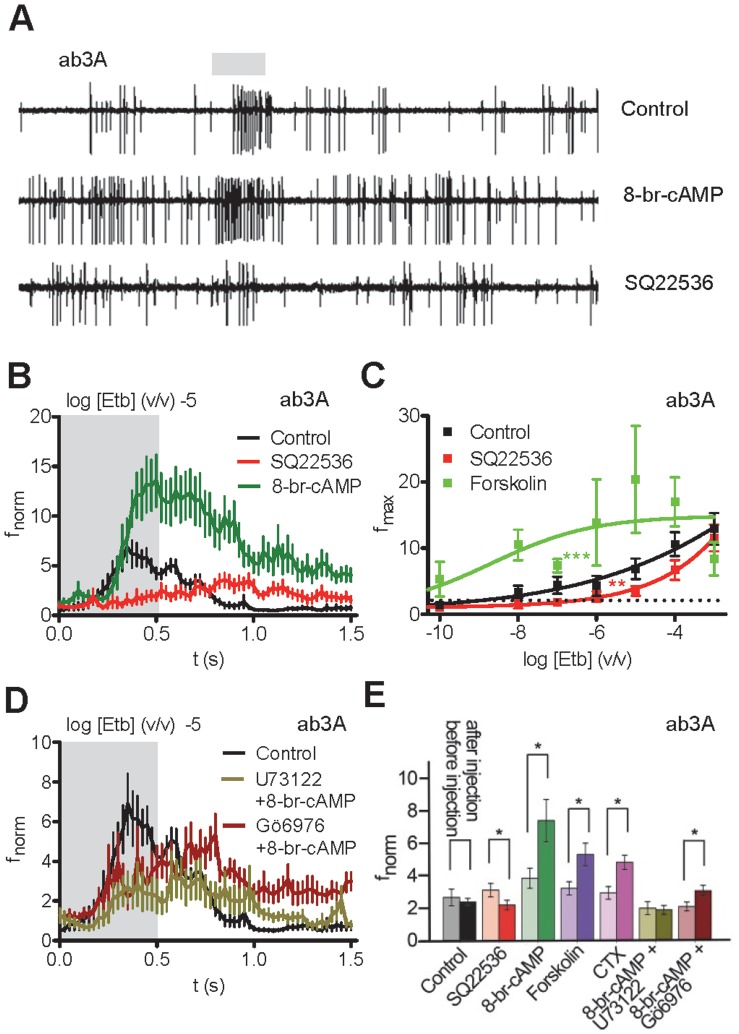
Manipulation of cAMP signalling in *Drosophila* ab3 sensilla affects the odorant response. **A**, Recordings of neuronal activity (large action potentials, Or22a neuron; small action potentials, Or85b neuron) before and after Etb stimulation (−5 v/v; 0.5 s, shaded area) in the presence of indicated compounds. While 8-br-cAMP enhances the Etb response, inhibition of adenylyl cyclase with SQ22536 attenuates it. **B**, Normalized spike frequency (f_norm_) of ab3A upon Etb stimulation (0 to 0.5 s, shaded area) at indicated dilution after injection of saline solution (Control; *n* = 11), of 8-bromo-cAMP (*n* = 11; *P*<0.05, Mann-Whitney U test) and of the adenylyl cyclase inhibitor SQ22536 (*n* = 17; *P*<0.01, U test). **C**, Concentration dependence of the maximum frequency f_max_ of f_norm_ to Etb stimulation after saline, forskolin and SQ22536 injection (**P<0.01, ***P<0.001, ANOVA). **D**, f_norm_ as described in (B) after injection of saline solution (Control; *n* = 11), U73122 plus 8-br-cAMP (*n* = 10; *P* = 0.18, U test), and Gö6976 plus 8-br-cAMP (*n* = 17; *P* = 0.16, U test). In the presence of the PLC or PKC inhibitors 8-br-cAMP fails to enhance the odor response. **E**, Comparison of treatment effects on Etb response before and after microinjection. f_norm_ on Etb stimulation (0.5 s) as determined from area under the curve measurements of the total response (1.35 s). Responses to Etb were measured 20 s after commencement of recording (before injection) and 200 s after injection (after injection) of the control (*n* = 11), SQ22536 (*n* = 17), 8-br-cAMP (*n* = 11), forskolin (*n* = 9; data from Olsson et al., 2011), cholera toxin (CTX; *n* = 12), 8-br-cAMP plus U73122 (*n* = 10), and 8-br-cAMP plus Gö6976 (*n* = 17). Error bars represent s.e.m. Asterisks indicate significant differences (*P*<0.05, Paired Wilcoxon Signed-Rank Test).

We then asked whether manipulation of intracellular signalling in Or-expressing OSNs could affect the odor response. Injection of the adenylyl cyclase inhibitor SQ22536 into the base of ab3A sensilla reduced the response to Etb (Fig. 3A–C). In contrast, injection of 8-bromo-cAMP, a membrane-permeable cAMP analog shown to activate OR dimers such as Or22a/Orco and Orco alone [9], enhanced the OSN response upon Etb stimulation (Fig. 3A, B). In line with this result, microinjection of the adenylyl cyclase activator forskolin enhanced the Etb response and shifted the concentration-dependence curve towards lower Etb concentrations (Fig. 3C). Taken together, inhibition of cAMP production weakened odor responses whereas enhancement of cAMP levels, either by direct injection or by adenylyl cyclase activation via forskolin or cholera toxin (Fig. 3E) augmented them.

The sensitivity of the Orco channel mediating this metabotropic response to cAMP is regulated by protein kinase C (PKC)-dependent phosphorylation [24]. Inhibition of phospholipase C (PLC) or PKC reduced the odor response in the fly whereas PKC activation enhanced it [24]. We thus asked whether inhibition of PLC or PKC could counteract the response potentiation by cAMP. Co-injection of 8-bromo-cAMP with the PLC inhibitor U73122 or the PKC inhibitor Gö6976 not only prevented any cAMP effect, but even diminished the Etb response with respect to the Control injection (Fig. 3D). The sensitivity of the odor response is thus influenced by secondary regulation of Orco channel activity.

### Regulation of OR Function is Intrinsic

**Figure 4:**
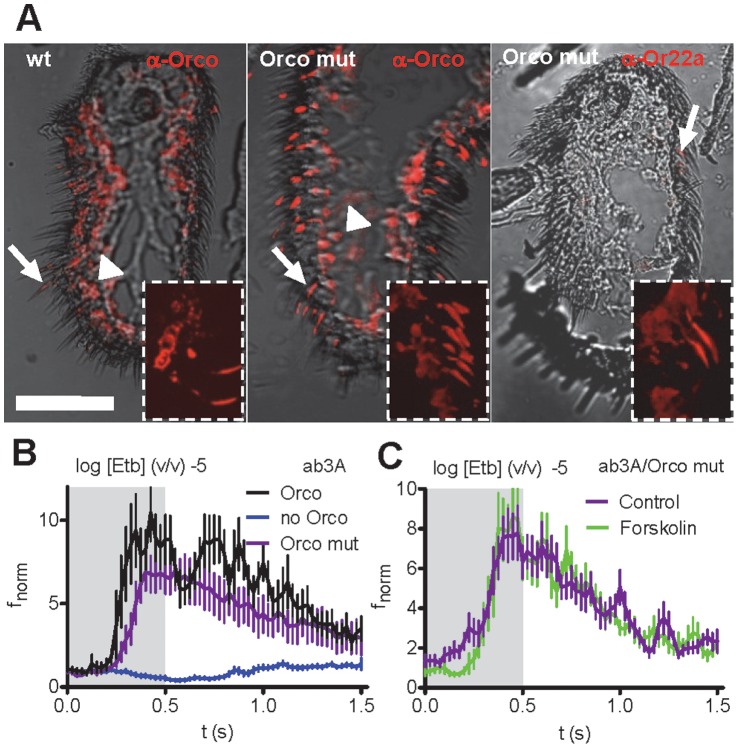
Regulation of OR response by cAMP signaling is intrinsic. **A**, Orco (left), Orco mut (middle) and Or22a (right) proteins visualized in adult antennal sections with specific antibodies (red). The proteins show expression in cell bodies (arrowhead) and dendrites (arrow). Or22a-expressing cells are housed in few sensilla opposite to arista (a). Scale bar 50 mm. **B**, Normalized ab3A neuron spike frequency (f_norm_) upon Etb stimulation wild type flies (Orco, *n* = 12), for Orco null mutants (no Orco, *n* = 15), and mutants rescued with Orco mut (“Orco mut flies”; *n* = 14; *P* = 0.016 vs. Control, Mann-Whitney U test). **C**, f_norm_ as in **B** upon Etb stimulation in Orco mut flies (*n* = 17) before (Control) and after forskolin injection.

Manipulation of intracellular signalling cascades may affect cellular targets other than ORs. Raising the cAMP concentration can, for example, activate cyclic nucleotide gated channels [33]. We thus inhibit Orco sensitivity to cAMP to assess whether the effect of intracellular signalling is intrinsic to the Or/Orco complex. The activation of Orco by cAMP requires a basal PKC-mediated phosphorylation [24]. We previously created an Orco mutant (called Orco mut) with excluded phosphorylation by S/T to N exchanges in all five PKC sites, which is virtually insensitive to cAMP [24]. By replacing the expression of Orco with Orco mut, we produced a fly line with an inactive metabotropic pathway. In Orco null mutant flies we rescued Orco or Orco mut (Fig. 4A) in all Or-expressing OSNs [31]. If our observed effect of intracellular signalling is extrinsic to the OR complex, then cAMP production should enhance the OR response even when Orco is insensitive to cAMP.

Antennal sections immunostained against Orco and Or22a (Fig. 4A) showed appropriate expression of Orco mut and Or22a proteins in the dendrites of “Orco mut flies”, indicating that the chaperone function of Orco required to transfer the odorant-specific OR proteins into the plasma membrane [31] was not affected in Orco mut flies. Accordingly, these OSNs also responded to odorant stimulation (Fig. 4B). Nevertheless, injection of forskolin into ab3 sensilla did not change the Etb response (Fig. 4C; f_norm_ = 4.17±0.43 before and 4.04±0.55 after injection at log −5 Etb; *P* = 0.41, paired Wilcoxon signed ranks test). To exclude a saturation of the odorant response at log −5 Etb in Orco mut flies, we also tested lower Etb concentrations. For log −6 Etb to log −8 Etb, forskolin injection also did not significantly change the maximum f_norm_ (Student’s t test). This indicates that forskolin injection, and therefore intracellular signalling, acts on the OR complex intrinsically.

### Orco Activation Sensitizes ORs and Orco Inactivation Prevents Sensitization

**Figure 5:**
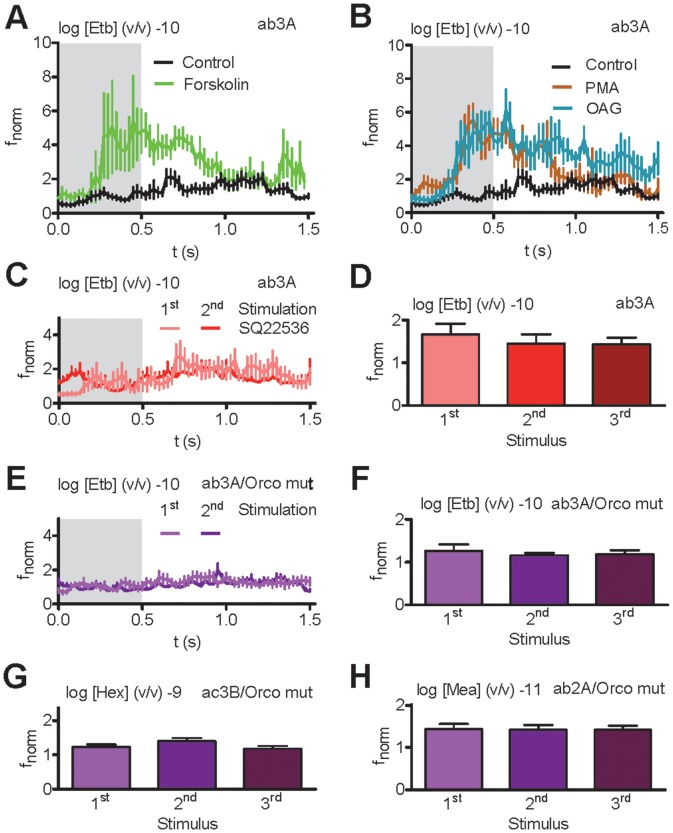
OR sensitization is mimicked by Orco activation and disrupted by Orco inhibition. **A**, **B**, f_norm_ for ab3A neurons expressing Or22a upon initial subthreshold Etb stimulations (log [Etb] −10) after injection of saline (Control;, forskolin (**A**, *n* = 8; *P*<0.05, Mann-Whitney U test), and the protein kinase C activators PMA (**B**, *n* = 7; *P*<0.001, U test) and OAG (**B**, *n* = 7; *P* = 0.001, U test). **C**, Time course of f_norm_ upon 1^st^ and 2^nd^ subthreshold Etb stimulation (log [Etb] −10, interval 20 s) after injection of SQ22536 (*n* = 13). **D**, Mean f_norm_ for Or22a neurons to repetitive subthreshold Etb stimulations (interval 20 s) after injection of SQ22536 (*n* = 13). **E**, Time course of f_norm_ for neurons expressing Orco mut upon 1^st^ and 2^nd^ subthreshold Etb stimulation (log [Etb] −10, interval 20 s, *n* = 12). **F – H**, Mean f_norm_ for ab3A (**F**), ac3B (**G**) and ab2A (**H**) neurons expressing Orco mut to repetitive subthreshold Etb (**F**), Eta (**G**) and Mea (**H**) stimulations (interval 20 s, *n* = 8–14). N.s.; Paired Wilcoxon Signed Ranks test.

As repetitive subthreshold odorant stimulation was seen to elicit an OSN response, we asked whether cAMP production could sensitize ORs (Fig. 1). Adenylyl cyclase stimulation via microinjection of forskolin prior to subthreshold Etb stimulation (log −10 dilution) of Or22a-expressing OSNs induced a response already at the initial odor pulse (Fig. 5A). A similar effect was observed upon PKC stimulation with OAG or PMA microinjection (Fig. 5B). Thus, activation of Orco through intracellular signalling sensitizes the OR to respond to subthreshold odor concentration.

Inhibition of adenylyl cyclase via SQ22536 prevented receptor sensitization (Fig. 5C, D), and repeated subthreshold Etb stimulations failed to elicit a response in Orco mut flies, further indicating that receptor sensitization requires metabotropic signalling (Fig. 5E, F). In these flies, the essential role of Orco function for OSN sensitization was also shown for ab1 sensilla housing Or42b expressing OSNs and ab2 sensilla with Or59b expressing OSNs (Fig. 5G, H).

It should be mentioned that, although injection of cAMP for 200 s strongly enhanced the Etb response (Fig. 3B), it did not increase the spontaneous activity of the ab3A neuron (Fig. S1B). Thus, the stimulation of the odor response by Orco activation need not be accompanied by Orco pacemaker activity.

## Discussion

Although both insect ORs and IRs operate as ionotropic receptors, their tuning properties differ fundamentally. While prolonged stimulation leads to adaptation of ORs, there is no adaptation of IRs [16]. On the other hand, ORs but not IRs expand their dynamic range through intrinsic sensitization. This difference in sensitization is apparent even between ORs and IRs expressed in co-localized sensilla (c.f. Fig. 1E, Fig. 2B–D). Thus, sensitization must result from intrinsic, rather than extrinsic neuronal properties that are unique to ORs. The most parsimonious explanation for the mechanistic differences between these families, is the use of intracellular signalling to modulate OR activity [34]. Given the previous *in vivo* evidence for a role of metabotropic signalling in OR function [21], [23], [35]–[38], we first pursue the metabotropic regulation of Orco in mediating OR activity.

OR sensitization could be mimicked by manipulations enhancing cAMP production or PKC activity and depressed by inhibition of cAMP production or PLC/PKC activity (Fig. 5). These intracellular signalling systems not only influence the OR sensitivity at weak odor stimuli, they also modulate the OR response for stronger stimuli (Fig. 3). In detail, microinjection of cAMP or adenylyl cyclase activators into sensilla increased the odorant response and shifted the dose-response curve toward lower odorant concentrations. A previous study has revealed that Orco sensitivity to cAMP is regulated by protein kinase C (PKC)-dependent phosphorylation [24]. Our results show that inhibition of PLC or PKC also inhibited any effect of cAMP, indicating that the enhanced sensitivity caused by cAMP is regulated by Orco activity. The metabotropic regulation of Orco also lead to sensitization of the OSN to repeated subthreshold odor responses, which is abolished by adenylyl cyclase inhibition. Furthermore, the sensitization of the odor response was blocked in mutant flies with impaired Orco phosphorylation (Orco mut) further indicating that metabotropic regulation of Orco activity is required for the enhanced odorant response. It cannot be excluded that cAMP and PKC activation may regulate OR sensitivity to odors via other mechanisms, such as through modulation of membrane traffick. Nevertheless, the lack of response modulation following injection of forskolin into PKC flies, indicates that the metabotropically-enhanced odor sensitivity is intrinsic to the OR complex and does not result from extrinsic cellular processes.

Our results thus suggest that intracellular signalling, and in particular metabotropic regulation of Orco, plays a vital role in conferring the mechanistic differences between ORs and IRs. Although we cannot yet confirm the mechanistic basis of intracellular signalling in these OSNs, we can conclude that modulations that activate Orco when heterologously expressed enhance the odor sensitivity of ORs *in vivo* and, vice versa, modulations that inhibit Orco reduce OR sensitivity. It must also be kept in mind that the ORs are Ca^2+^-permeable, constitutively active ion channels [8], [9], the background activity of which is also able to activate enzymatic activity. Future studies should characterize the composition of the respective signalling subsystems, e.g. those involved in sensitizing receptors vs. those involved in terminating the odorant response.

The evolution of a highly sensitive and adaptable olfactory system is believed to be a key factor allowing insects to radiate into more or less every environment on earth [2]. Given the importance of OSN dynamics in tracking turbulent odor plumes [39], olfactory sensitization via Orco regulation can enhance an insect’s ability to accurately detect and respond to intermittent, low concentration stimuli [16]. Insect ORs are thought to have evolved from ionotropic gustatory receptors [40], which detect millimolar ligand concentrations [41]. Our results imply that the special heterodimeric design of ORs has likely evolved to quickly detect and respond to volatile compounds at very low concentrations, such as those encountered by flying insects. Regardless of the source of this difference, it is clear that the OR expansion of ionotropic receptors offers the insect olfactory system both broadened ligand affinity as well as expanded spatiotemporal dynamics with which to navigate the olfactory world.

## Acknowledgments

We thank S. Bucks, S. Dietel, S. Kaltofen, R. Schäfer and R. Stieber for technical assistance, and L. Vosshall for providing antibodies.

## Supporting Information

Figure S1 **Effect of compound injection on spontaneous activity of OSNs.** **A**, Recordings of spontaneous spike frequency (f_norm_, normalized to first 15 s of recording) for ab3A and ab3B neurons with injection of saline (Control, ab3A, *n* = 5) or ethyl butyrate (Etb, ab3A, ab3B, *n* = 11) at 100 s. **B**, Recordings of f_norm_ for ab3A neurons with injection of saline (Control, *n* = 5) or 8-br-cAMP (*n* = 12) at 100 s.
(TIF)
