# Supplementary figures and images for: Insect Odorant Response Sensitivity Is Tuned by Metabotropically Autoregulated Olfactory Receptors
Source: PLoS One. 2013 Mar 12;8(3):e58889. doi: 10.1371/journal.pone.0058889 (PMC3595248; doi:10.1371/journal.pone.0058889)

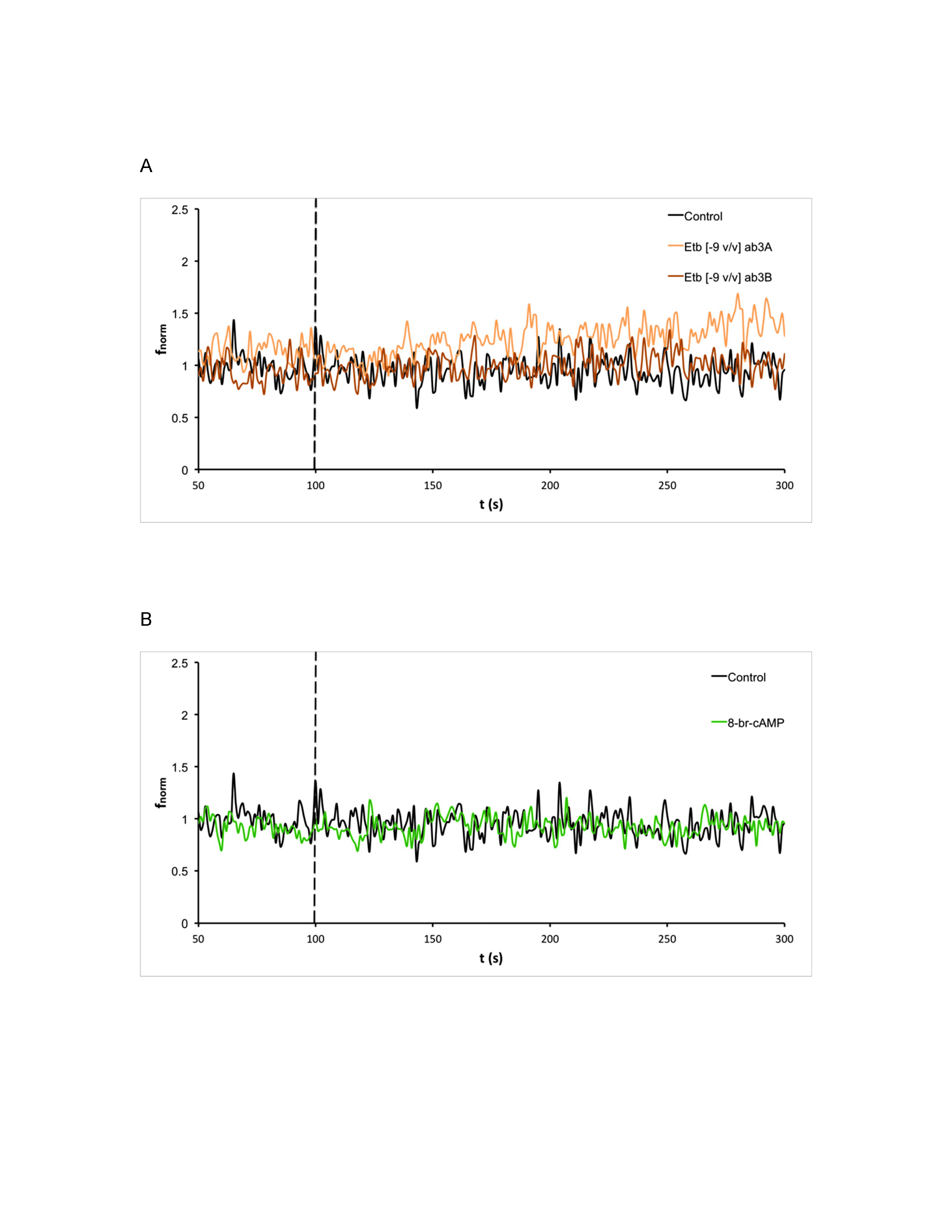

Supplement: Figure S1 — Effect of compound injection on spontaneous activity of OSNs. A, Recordings of spontaneous spike frequency (fnorm, normalized to first 15 s of recording) for ab3A and ab3B neurons with injection of saline (Control, ab3A, n = 5) or ethyl butyrate (Etb, ab3A, ab3B, n = 11) at 100 s. B, Recordings of fnorm for ab3A neurons with injection of saline (Control, n = 5) or 8-br-cAMP (n = 12) at 100 s. (TIF) [file pone.0058889.s001.tif]
